# Supplementary material for: Impact of Introducing Hepatitis B Birth Dose Vaccines into the Infant Immunization Program in Burkina Faso: Study Protocol for a Stepped Wedge Cluster Randomized Trial (NéoVac Study)
Source: Vaccines (Basel). 2021 Jun 1;9(6):583. doi: 10.3390/vaccines9060583 (PMC8227098; doi:10.3390/vaccines9060583)
Supplement: Supplementary file 1 [file vaccines-09-00583-s001.zip › Document S1. Patient Information Sheet.pdf]

**NEOVAC 2 - Burkina Faso - Doc01**

*« Evaluation de l'intégration d'une dose néonatale de vaccin contre l'hépatite B dans le calendrier vaccinal des enfants au Burkina Faso une méthode mixte comprenant un essai communautaire, une étude anthropologique et une évaluation économique ».*

Promoteur : Institut Pasteur, 25-28 rue du Docteur Roux, 75015 Paris

En tant que promoteur, l'Institut Pasteur en collaboration avec le LAMIVAC mène une étude sur l'hépatite B au Burkina Faso, dans les districts sanitaires de Do et de Dafra.

A l'occasion d'une visite prénatale ou lors de votre accouchement, il vous a été proposé de participer à cette étude en faisant vacciner votre enfant contre l'hépatite B dès sa naissance. Vous êtes libre d'accepter ou de refuser de participer à cette étude.

Cette étude est financée par la Fondation Total. Les laboratoires Gilead et Abbott contribuent également au financement de ce projet.

**Qu'est-ce que l'hépatite B ?**

L'hépatite B est une infection qui est très répandue en Afrique sub-Saharienne.

Cette infection peut être responsable du développement d'une cirrhose ou d'un cancer du foie, cause de décès à l'âge adulte. Elle peut être évitée par la vaccination.

Il existe une vaccination contre l'hépatite B pour les enfants dans le cadre du Programme Elargi de Vaccination (PEV), celle-ci débute à l'âge de 2 mois et protège les enfants contre la transmission du virus par contact avec d'autres personnes infectées dans la petite enfance.

**Quel est l'objectif de cette étude ?**

Le but de cette étude est de montrer l'efficacité de la vaccination contre l'hépatite B en commençant dès la naissance pour protéger l'enfant directement après l'accouchement, de vérifier si cette vaccination est faisable et acceptable auprès de vous et des personnels de santé et si son coût pour l'Etat n'est pas trop élevé.

A terme, cela permettrait de faire bénéficier de cette vaccination à tous les enfants nés au Burkina Faso.

**Pourquoi vacciner à la naissance ?**

Le vaccin à la naissance permet de protéger les enfants des risques de contamination entre la maman et le bébé. Cette transmission peut avoir lieu pendant la grossesse ou lors de l'accouchement quand la maman est infectée.

**Est-ce recommandé par les autorités de santé d'administrer un vaccin contre l'hépatite B à la naissance ?**

L'Organisation Mondiale de la Santé (OMS) encourage la vaccination contre l'hépatite B dans les 24 heures suivant la naissance d'un enfant. Elle est recommandée au Burkina Faso mais elle n'est pas encore mise en place dans les Centres de Santé et de Promotion Sociale (CSPS).

**Comment se déroule cette étude ?**

Cette étude impliquera tous les CSPS ruraux des districts sanitaires de Dafra et de Dô, soit environ 8500 couples mère/enfant sur une durée de deux ans.

Dans le cadre de cette étude, les CSPS ne proposeront pas la vaccination des enfants à la naissance tous en même temps. Ceux-ci commenceront à vacciner, au fur et à mesure après avoir été tirés au sort. Chaque mois, un nouveau CSPS pourra débuter la vaccination à la naissance.  Il ne sera donc pas possible de savoir si votre CSPS vaccinera ou non avant votre accouchement.

**Qu'est-ce que cette étude implique pour moi et mon futur enfant ?**

Dans un premier temps : questionnaire

Si vous acceptez de participer à cette étude vous serez invitée à répondre à un questionnaire sur vous et votre foyer. Les questions porteront notamment sur votre situation matrimoniale, le nombre d'accouchements dans votre vie et votre niveau d'instruction. Cette première visite aura lieu au cours d'une consultation prénatale ou lorsque vous viendrez accoucher au CSPS.

Dans un second temps : accouchement

Votre participation implique de venir accoucher dans le CSPS dans lequel vous avez effectué vos visites prénatales.

Si votre CSPS dispose des vaccins et que vous avez accepté de participer à l'étude, votre enfant sera vacciné contre l'hépatite B dès sa naissance.

Dans le cas où le vaccin à la naissance n'a pas été mis en place dans votre CSPS au moment de votre accouchement, votre enfant sera vacciné à 2 mois dans le cadre du Programme Elargi de Vaccination (PEV).

Au cours de cette visite, que votre enfant soit vacciné à la naissance ou non, nous recueillerons des données en lien avec votre grossesse et votre accouchement ainsi que sur votre enfant.

Dans un troisième temps : prélèvement de sang et questionnaire

Votre participation implique aussi de prélever quelques gouttes de sang au bout de votre doigt et de celui de votre enfant pour réaliser un test de dépistage de l'hépatite B. Vous serez directement informée du résultat de ce test.

Cette petite piqûre, non douloureuse, pourra être faite à l'occasion de la consultation du PEV au cours de laquelle votre enfant recevra ses vaccins.

Si le résultat du test est positif chez vous ou votre enfant, cela veut dire que vous ou votre enfant êtes infectés par le virus de l'hépatite B (rare). Nous réaliserons alors une prise de sang pour des examens de laboratoire plus approfondis. Dans le cas où une prise de sang doit être réalisée chez votre enfant, il vous sera proposé de lui appliquer un patch anesthésiant et/ou de le prélever au cours de l'allaitement pour qu'il ne ressente pas une éventuelle douleur lors de l'insertion de l'aiguille.
Ces résultats nous permettrons de vous orienter dans les meilleurs délais et dans les meilleures conditions pour prendre en charge votre maladie ou celle de votre enfant.

Nous recueillerons aussi des données sur les visites postnatales de votre enfant et ses vaccinations ainsi que les données biologiques des prélèvements sanguins réalisés.

Analyses génétiques :

Enfin, pour nous permettre de comprendre pourquoi certaines personnes développent une maladie chronique lors d'une infection par le virus de l'hépatite B alors que d'autres guérissent spontanément, nous souhaiterions avoir la possibilité de réaliser des analyses génétiques à partir de vos échantillons sanguins ou ceux de votre enfant. Ces analyses pourraient permettre de trouver quels gènes sont associés au risque de développer l'hépatite B. Les analyses génétiques se limiteront à la recherche de gènes de susceptibilité à l'hépatite B.

La durée totale de votre participation sera d'environ 10 mois, période qui couvre la dernière visite prénatale jusqu'à la visite du PEV à 9 mois. Mais sachez que vous avez le droit de mettre fin à votre participation à tout moment de l'étude (1$^{er}$, 2$^{nd}$ et 3$^{ème}$ temps) sans vous justifier et sans que cela ait des conséquences négatives

sur la qualité de votre suivi. Vous pouvez tout à fait consentir à participer et répondre aux questions puis refuser finalement de vacciner votre enfant à la naissance. L'équipe médicale a l'obligation de vous suivre et ne changera pas ses pratiques du fait de votre refus ou de votre changement d'avis. C'est très important.

Si votre CSPS dispose des vaccins mais que vous ne souhaitez pas que votre enfant soit vacciné à la naissance, **vous pouvez quand même participer à l'étude** en répondant aux questionnaires et en acceptant le dépistage de l'hépatite B pour votre enfant et vous-même à la visite des 9 mois.

**Quel est le devenir des prélèvements ?**

Les papiers buvards contenant quelques gouttes de votre sang et de celui de votre enfant seront récupérés et conservés dans le laboratoire du Centre MURAZ à Bobo-Dioulasso. Des analyses biologiques dans le domaine des hépatites virales pourront être réalisées dans un second temps localement ou dans d'autres pays selon les moyens techniques disponibles.

Pour les mères et les enfants qui seront dépistés positifs pour l'hépatite B, leurs échantillons de sang seront transportés au Centre MURAZ pour y être analysés afin de connaître le niveau du virus dans le sang et l'état du foie. Ces résultats seront alors transmis à un médecin généraliste du Centre Médical avec Antenne chirurgicale (CMA) de votre district et nous vous inviterons à le consulter pour votre prise en charge ou celle de votre enfant. Une petite partie de l'échantillon de sang sera conservée au Centre Muraz pendant 15 ans.

D'autres analyses biologiques dans le domaine des hépatites virales, incluant des analyses génétiques, pourront être réalisées dans un second temps localement ou dans d'autres pays selon les moyens techniques disponibles. Dans le cadre de ces analyses complémentaires, vos échantillons et données pourront être envoyés à des partenaires académiques ou industriels du projet.

**Quel est le bénéfice de ma participation et celle de mon enfant ?**

Votre enfant bénéficiera peut-être d'une vaccination à la naissance contre l'hépatite B, cependant, si ce n'est pas le cas, il débutera sa vaccination contre l'hépatite B à l'âge de 2 mois et poursuivra ses vaccinations comme tous les autres enfants pris en charge dans les CSPS au Burkina Faso.

A 9 mois, vous et votre enfant bénéficierez d'un test gratuit pour savoir si vous êtes infectés ou non par le virus de l'hépatite B.
En cas de dépistage négatif pour vous, cela signifie que vous n'êtes pas atteinte par la maladie, soit parce que vous n'avez jamais été en contact avec le virus (ce qui est rare au Burkina Faso) soit parce que vous avez été en contact avec le virus mais que vous vous en êtes débarrassée et que vous êtes donc guérie (situation la plus fréquente au Burkina Faso). Afin de confirmer ce point, il est conseillé de réaliser un test et si vous n'avez jamais été en contact avec le virus vous pourrez vous faire vacciner contre l'hépatite B par le personnel de l'étude.
En cas de dépistage positif, vous ou votre enfant serez orientés vers des médecins pour votre prise en charge et le traitement de la maladie si nécessaire. Les examens complémentaires qui seront réalisés permettront au médecin d'établir s'il est nécessaire de vous prescrire un traitement contre l'hépatite B ou pas. En cas de dépistage positif, les consultations, examens et médicaments seront pris en charge par l'étude pendant une durée de 2 ans.

Les résultats de vos tests permettront de savoir si le vaccin et la stratégie adoptée pour vacciner à la naissance sont efficaces pour prévenir les infections entre la mère et l'enfant autour de la naissance. Les connaissances acquises à travers cette étude pourront bénéficier à la santé publique du Burkina Faso et plus largement à l'Afrique.

Si vous le souhaitez, vous pourrez être informée des résultats globaux de la recherche par l'intermédiaire du personnel de l'étude.

**Quels sont les risques ?**

De manière générale, seules des réactions indésirables minimes, telles que gonflement au point d'injection, douleurs localisées, douleurs musculaires ou fièvre transitoire, sont observées, la plupart du temps dans les 24 heures suivant la vaccination. Ces réactions bénignes ont tendance à être moins fréquentes chez l'enfant que chez l'adulte. Dans des cas très rares, une réaction allergique grave peut survenir après une vaccination (1 cas pour 1 million). Si votre enfant présente des symptômes inhabituels après avoir été vacciné, il vous sera demandé de revenir au CSPS pour une consultation avec le personnel de santé.

Les rares risques liés à la prise de sang sont la survenance d'un hématome local, malaise vagal, léger saignement, douleur au moment de l'insertion de l'aiguille.

**Quels sont mes droits ?**

Votre participation à cette étude est entièrement volontaire, rien ne vous oblige à y participer. Vous pouvez prendre le temps de réfléchir et d'en parler avec votre conjoint ou votre famille avant de vous décider. Vous pouvez poser toutes les questions que vous souhaitez au personnel de l'étude.

Si vous décidez d'interrompre votre participation à la recherche, aucune nouvelle donnée vous concernant ne sera collectée ni ajoutée dans une base de données existante. Nous vous demanderons alors si vous nous autorisez à conserver les données déjà recueillies vous concernant durant votre participation à l'étude ainsi que vos échantillons biologiques ou si vous demandez leur destruction. Si nous ne parvenons pas à vous joindre, vos échantillons biologiques et les données associées, à l'exception des données de sécurité, seront détruits.

Nous vous rappelons que vous pouvez vous retirer de l'étude à tout moment sans avoir à vous justifier et sans conséquence pour vous ou votre enfant.

**Confidentialité des données**

Les informations que vous nous donnerez et qui seront analysées resteront strictement confidentielles. Votre nom ne sera indiqué nulle part : vos données seront identifiées par un numéro de code qui garantira la protection de vos données personnelles. Des explications complémentaires concernant vos données vous sont fournies dans le document d'information qui vous est remis avec cette note d'information.

**Cadre réglementaire**

L'Institut Pasteur, promoteur de cette recherche, a souscrit une assurance de responsabilité civile en cas de préjudice conformément aux dispositions du Code de la Santé Publique, auprès de la Société Nationale d'Assurances et de Réassurances, dont l'adresse est 284, avenue de Loudun – Ouagadougou – 01 BP. 406 (contrat n° 10023210000030).

Cette étude a reçu l'avis favorable du Comité d'Ethique pour la Recherche en Santé le 04/12/2018 et l'autorisation du Comité Technique d'Evaluation des Essais Cliniques le 04/11/2019.

Si vous êtes d'accord pour participer, merci de bien vouloir dater et signer le formulaire de consentement qui suit.

**Vous pouvez à tout moment faire valoir vos droits et demander des informations complémentaires auprès**

Du Médecin Chef du District Dô : **Le Docteur Ghislain Bouda Tél : 71 81 74 40 email : ghislainbouda@yahoo.com**
Du Médecin Chef du District Dafra : **Le Docteur Désiré CONGO Tél : 70754411 email : congodesire@yahoo.fr**
Du Coordonnateur de l'étude au LAMIVAC : **Le Docteur Abdoul TIENDREBEOGO Tél : 71120506 email : tiendraseric@gmail.com**
