# Supplementary material for: Impact of Introducing Hepatitis B Birth Dose Vaccines into the Infant Immunization Program in Burkina Faso: Study Protocol for a Stepped Wedge Cluster Randomized Trial (NéoVac Study)
Source: Vaccines (Basel). 2021 Jun 1;9(6):583. doi: 10.3390/vaccines9060583 (PMC8227098; doi:10.3390/vaccines9060583)
Supplement: Supplementary file 1 [file vaccines-09-00583-s001.zip › Document S2. Illustrated Patient Information Sheet.pdf]

LIVRET D'INFORMATION

ÉTUDE NÉOVAC 2 AU BURKINA FASO

ÉVALUATION DE L'INTÉGRATION D'UNE DOSE NÉONATALE DE VACCIN CONTRE L'HÉPATITE B DANS LE CALENDRIER VACCINAL DES ENFANTS AU BURKINA FASO UNE MÉTHODE MIXTE COMPRENANT UN ESSAI COMMUNAUTAIRE, UNE ÉTUDE ANTHROPOLOGIQUE ET UNE ÉVALUATION ÉCONOMIQUE

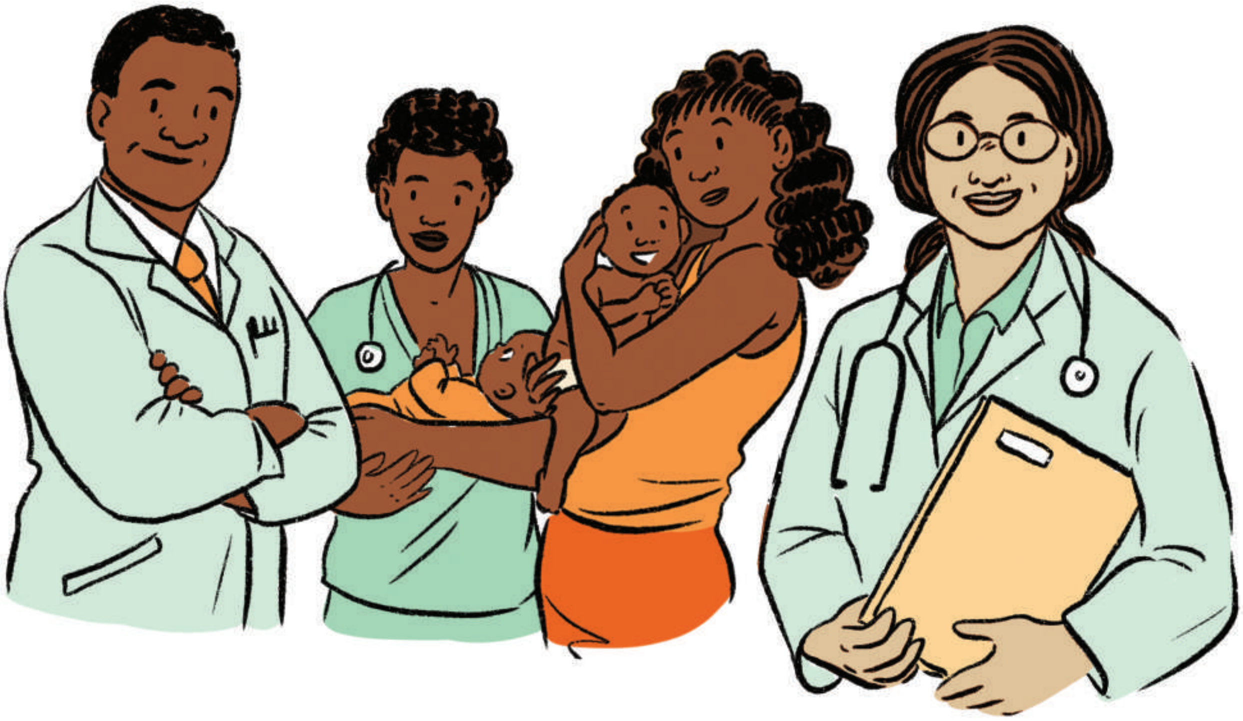

LAMIVAC

INSTITUT PASTEUR

L'HÉPATITE B

L'hépatite B est un virus.
Il est tellement petit qu'on ne peut pas le voir à l'oeil nu,
mais il peut se cacher dans le corps.

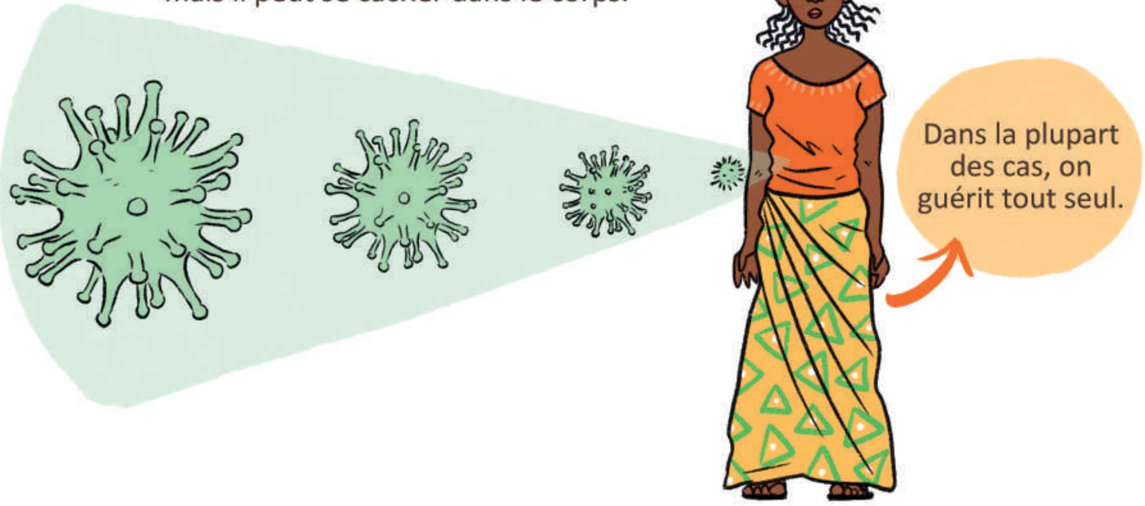

Mais chez certaines personnes, avec le temps, il peut créer des maladies graves.

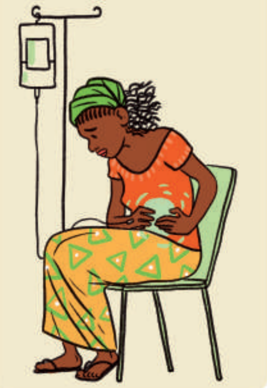

HEUREUSEMENT, IL EXISTE UN VACCIN !

Dans l'environnement, il y a beaucoup de microbes qui peuvent rendre malade.

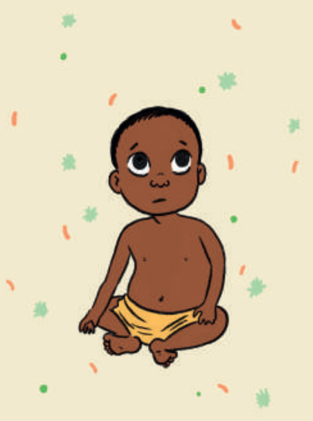

La vaccination protège le corps contre la maladie.

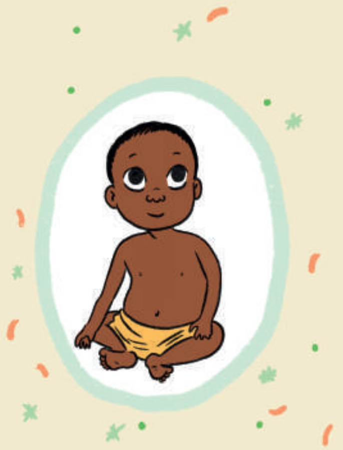

Une fois vacciné contre le virus, celui-ci ne peut plus nous rendre malade.

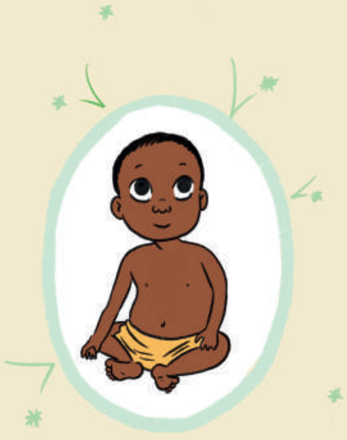

Pour l'instant, au Burkina Faso, la vaccination contre l'hépatite B débute à l'âge de 8 semaines.

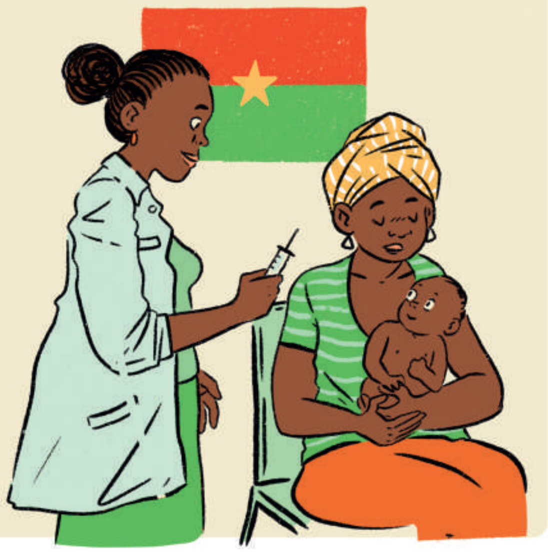

L'Organisation Mondiale de la Santé recommande de vacciner les enfants contre l'hépatite B à la naissance. Le vaccin est recommandé au Burkina Faso, mais pas encore mis en place.

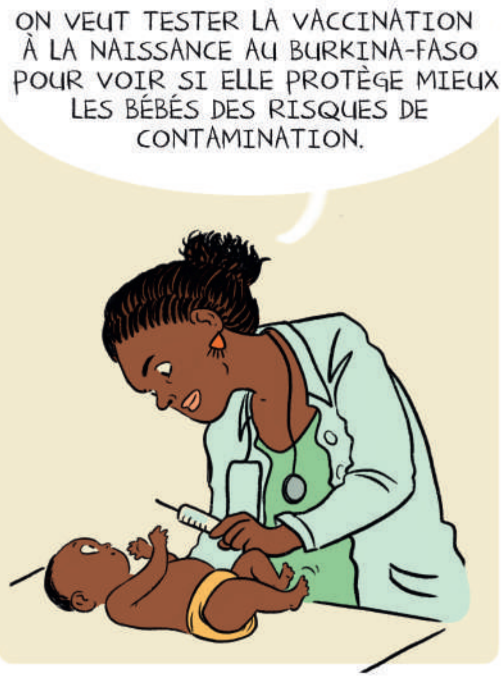

OÙ ?

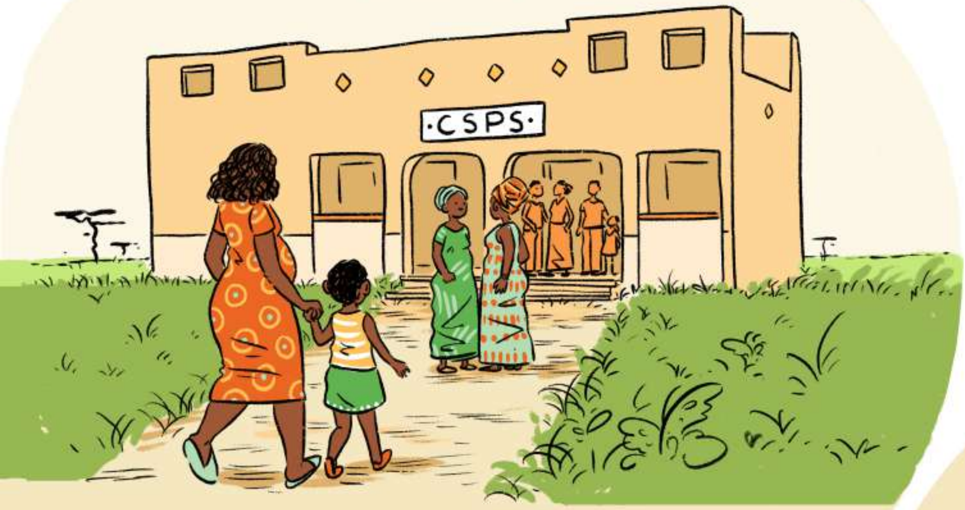

Chaque mois de nouveaux CSPS ruraux de Dafra et de Dô recevront le vaccin pour faire la vaccination à la naissance.
À la fin de l'étude, tous le feront.

2 possibilités :

Votre CSPS **fait** la vaccination à la naissance, votre enfant pourra être vacciné.

Votre CSPS **ne fait pas** la vaccination à la naissance.
votre enfant pourra être vacciné à 8 semaines.

QUI ?

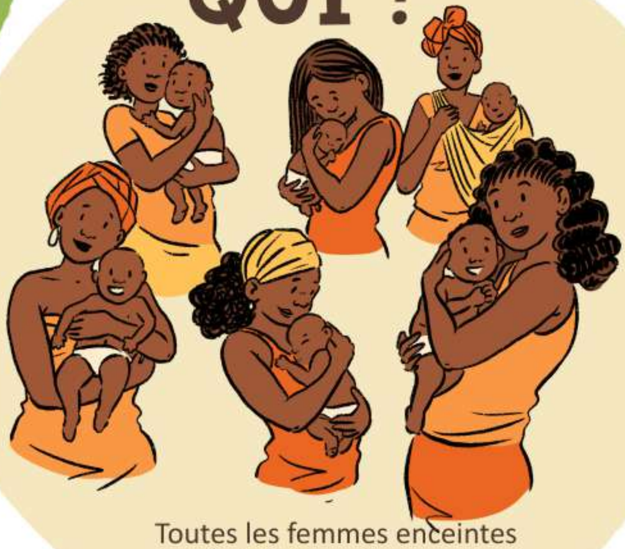

Toutes les femmes enceintes de Dô et Dafra qui accouchent dans les CSPS.

Visite prénatale

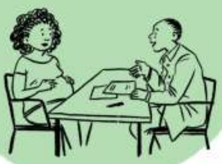

PLANNING

La durée totale de votre participation sera d'environ 10 mois.

Accouchement

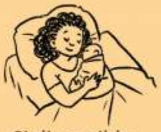

Si disponible, une dose de vaccin Hépatite B supplémentaire & Vaccination habituelle

Vaccination habituelle (Mois 2)

Vaccination habituelle (Mois 3)

Vaccination habituelle (Mois 4)

Test Hépatite B & vaccination habituelle (Mois 9)

Mois 1 | Mois 2 | Mois 3 | Mois 4 | Mois 5 | Mois 6 | Mois 7 | Mois 8 | Mois 9

QU'EST-CE QUE ÇA IMPLIQUE POUR MOI ET MON FUTUR ENFANT ?

PREMIER TEMPS
questionnaire au CSPS

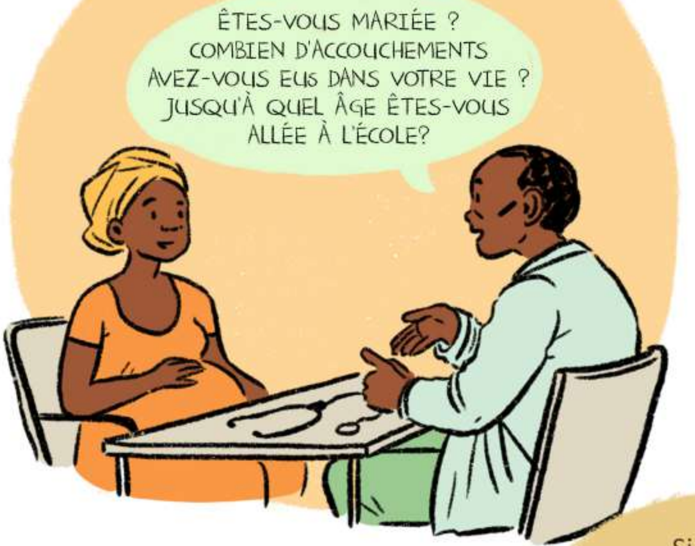

SECOND TEMPS
accouchement dans le même CSPS

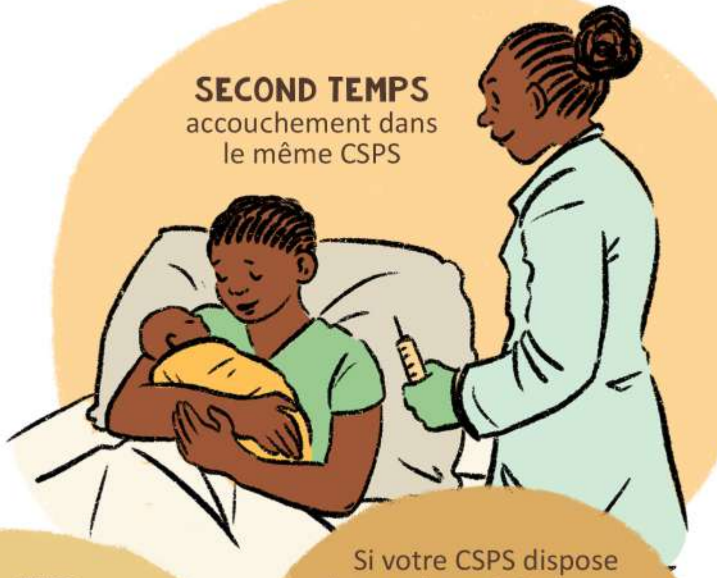

Si votre CSPS ne dispose pas de vaccins
↓
Vaccination habituelle & Vaccination Hépatite B à 2, 3 et 4 mois dans le cadre du Programme Elargi de Vaccination (PEV).

Si votre CSPS dispose des vaccins, et si vous avez accepté de participer à l'étude
↓
Vaccination habituelle & Vaccination hépatite B dès la naissance.

TROISIÈME TEMPS
prélèvement de sang et questionnaire

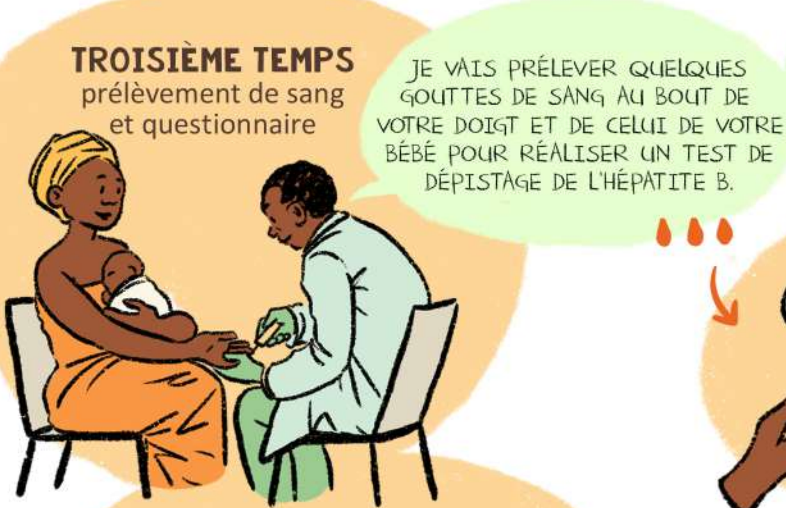

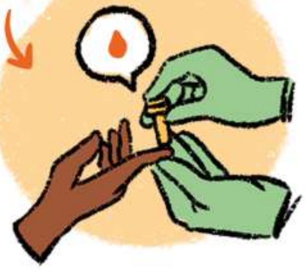

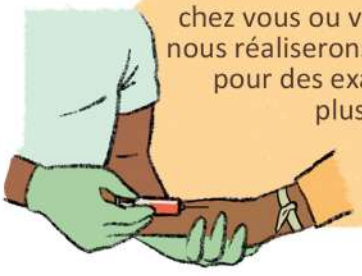

Si le résultat du test est POSITIF chez vous ou votre enfant (c'est rare), nous réaliserons alors une prise de sang pour des examens de laboratoire plus approfondis.

Si besoin de soins, vous serez pris en charge dans les meilleures conditions au Centre médical avec antenne chirurgicale.

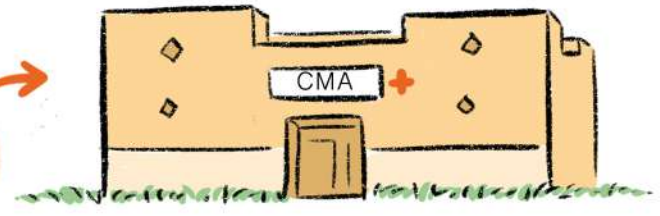

BÉNÉFICES

Si le vaccin est mis
en place dans votre CSPS,

Vaccination à
la naissance.

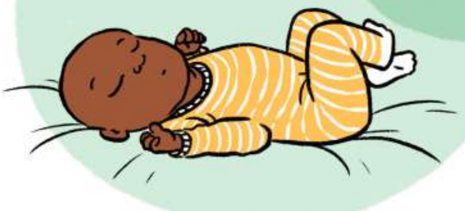

À 9 mois, test gratuit
de l'hépatite B pour vous
et votre enfant.

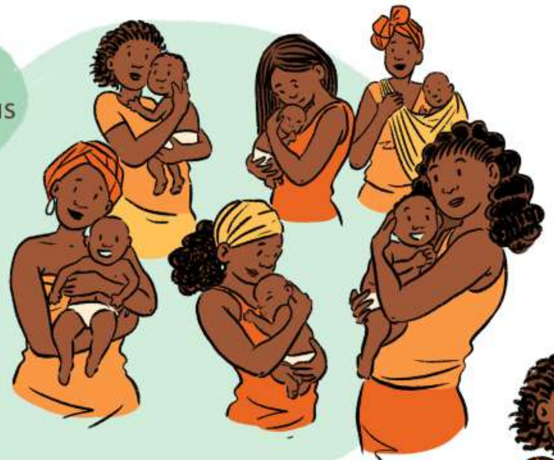

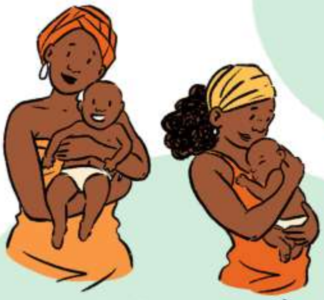

Si on est infecté et si
un traitement est nécessaire,
prise en charge pendant 2 ans.

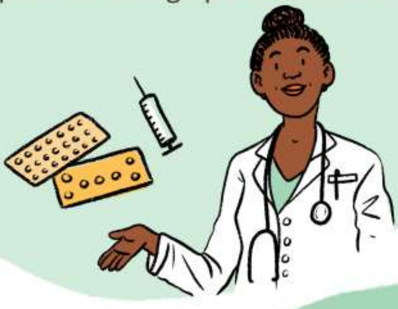

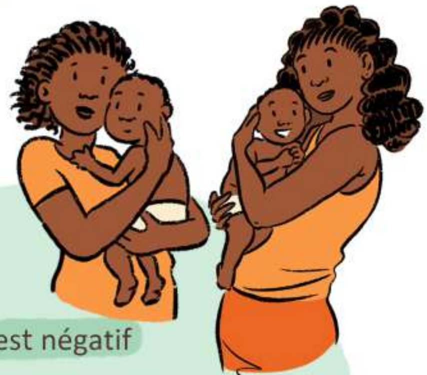

Si le test est négatif

Il est conseillé de réaliser un test
pour savoir si vous avez déjà rencontré
l'hépatite B et guéri toute seule.
Si vous n'avez jamais été en contact
avec le virus nous pourrons vous
vacciner contre l'hépatite B.

Aider la recherche pour :

Savoir si la stratégie de vaccination
à la naissance est efficace pour
prévenir les infections mère-enfant
autour de la naissance.

Améliorer la santé publique au
Burkina Faso et en Afrique.

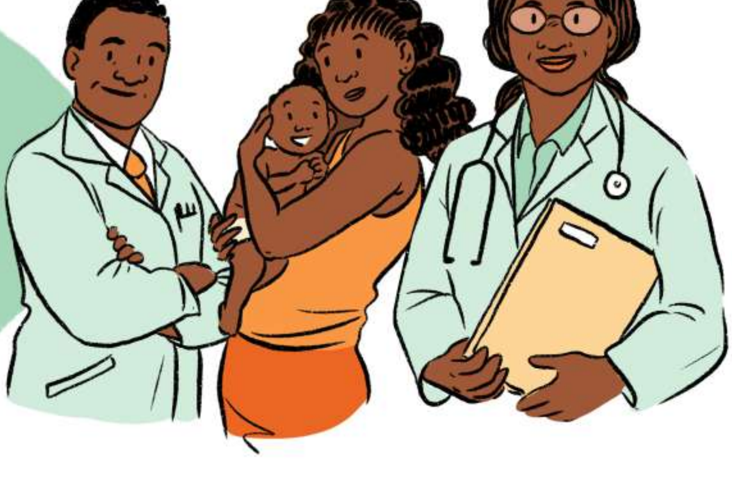

RISQUES

Parfois, dans les 24 heures
après la vaccination :

- Douleur, rougeur au point d'injection
- Diarrhées ou vomissements
- Fièvre

Mais, en général, ces effets disparaissent
rapidement.

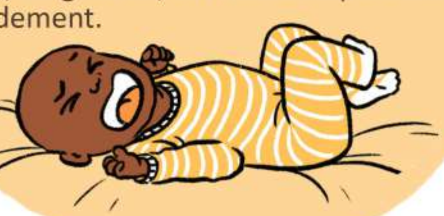

Très très rare
(1 cas pour 1 million) :
réaction allergique
grave.

VOS DROITS

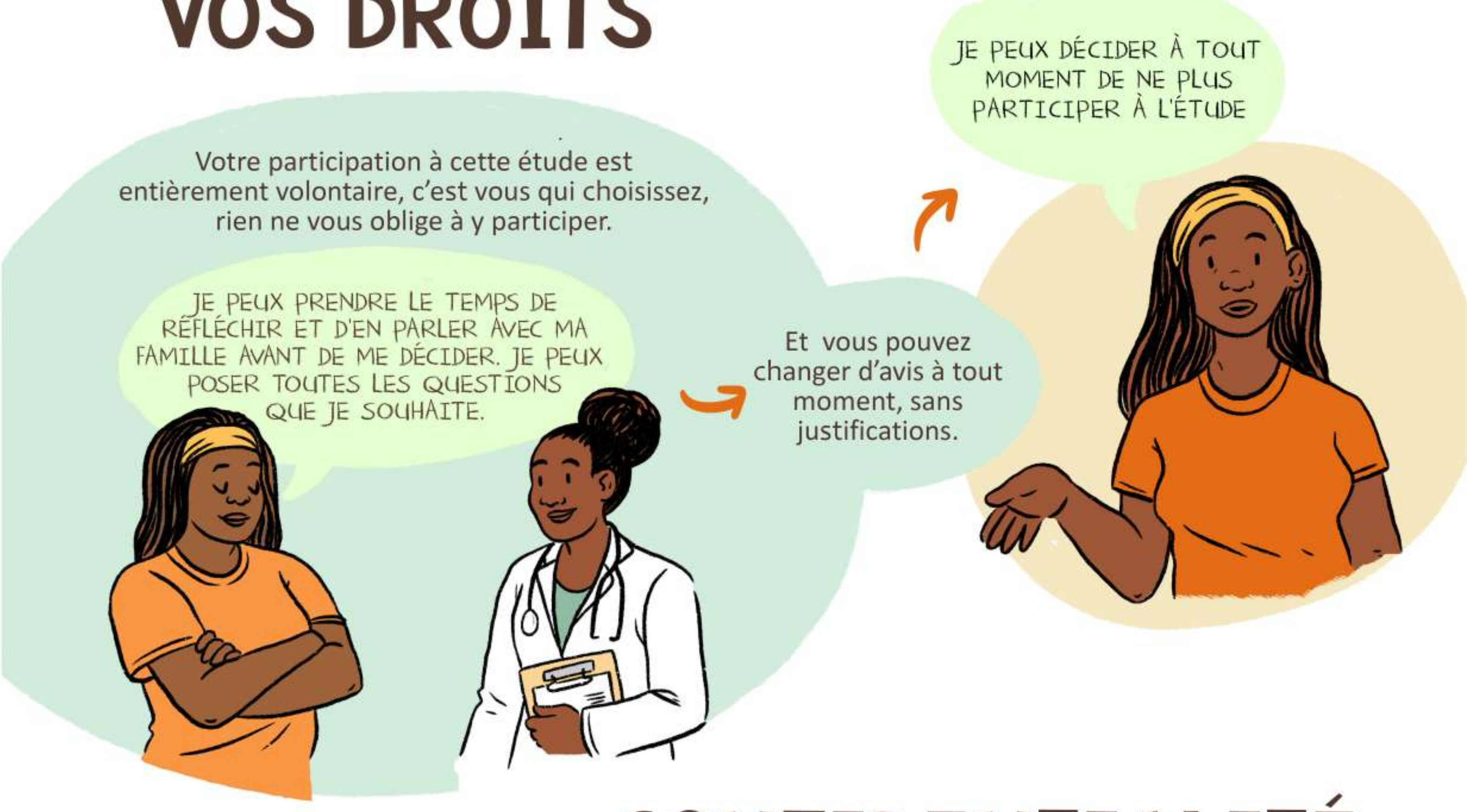

CONFIDENTIALITÉ DES DONNÉES

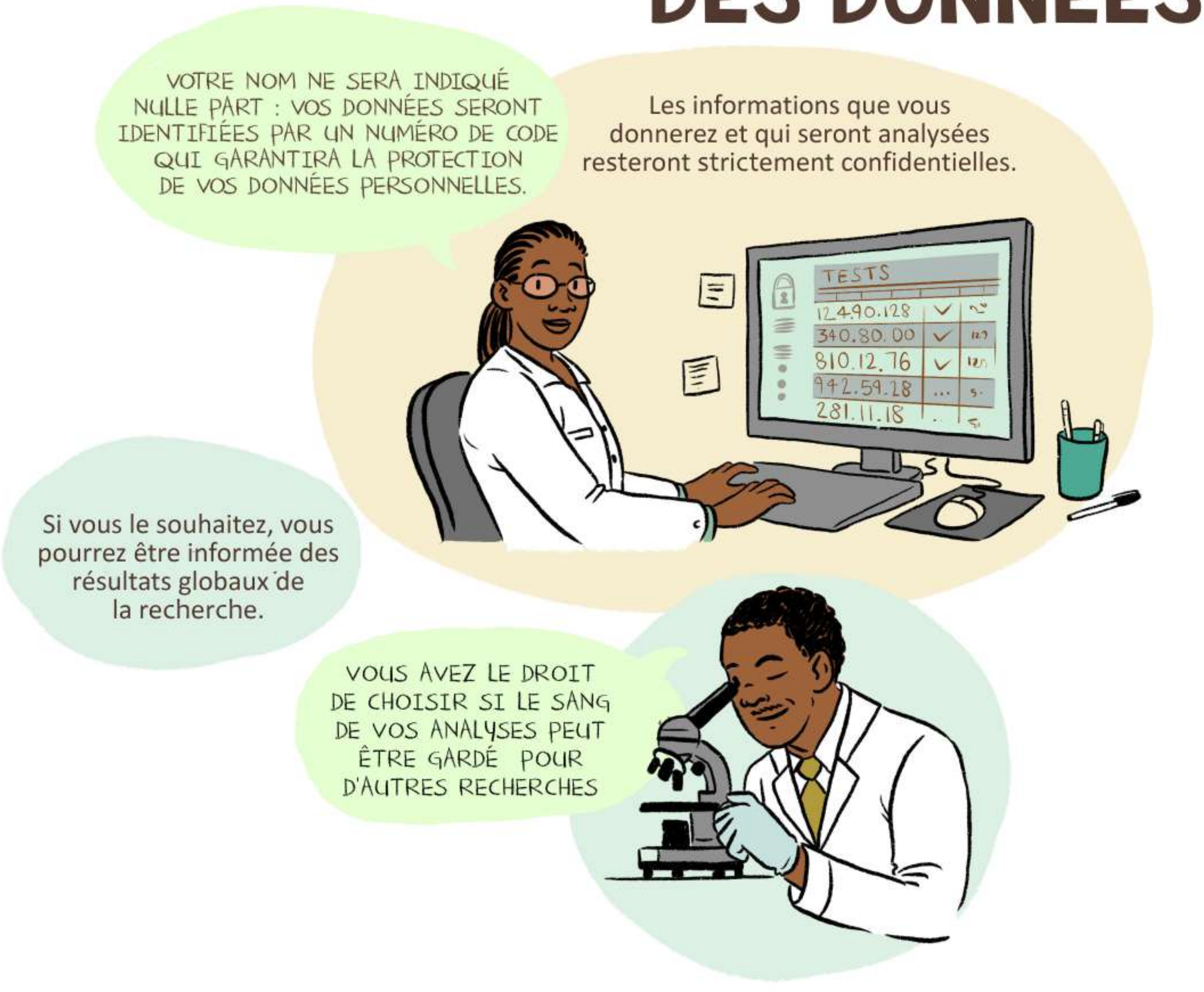

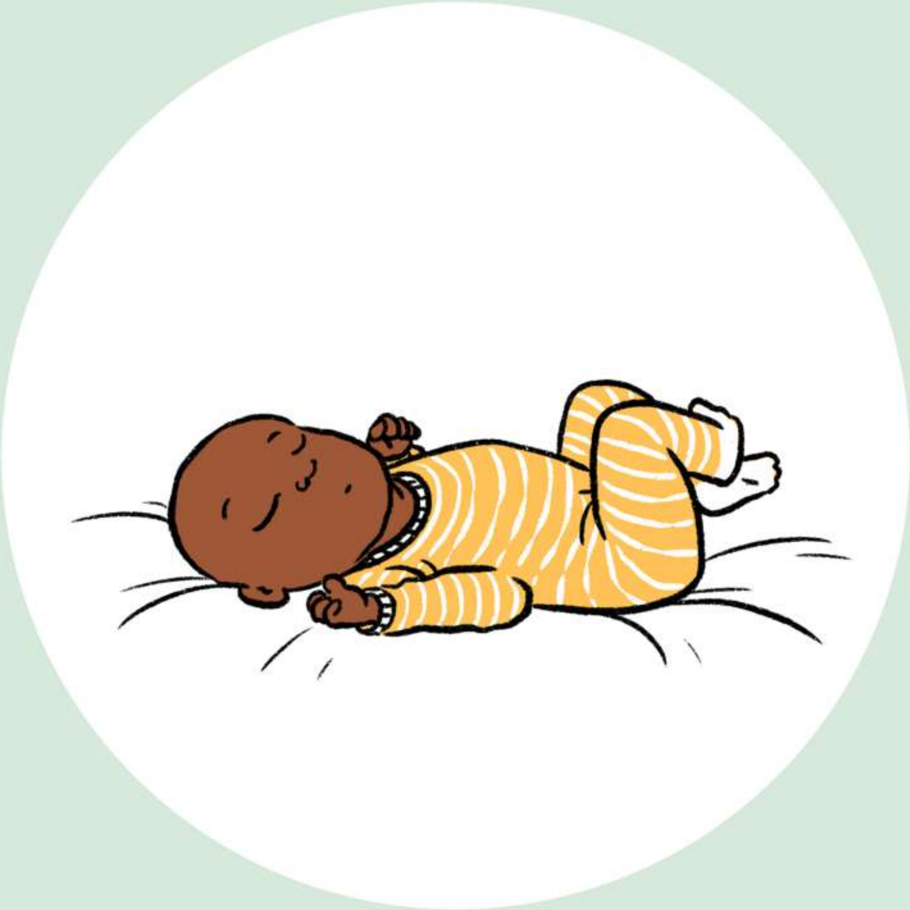

VOUS POUVEZ DEMANDER DES INFORMATIONS ET FAIRE VALOIR VOS DROITS AUPRES DE
