# Supplementary material for: Impact of Introducing Hepatitis B Birth Dose Vaccines into the Infant Immunization Program in Burkina Faso: Study Protocol for a Stepped Wedge Cluster Randomized Trial (NéoVac Study)
Source: Vaccines (Basel). 2021 Jun 1;9(6):583. doi: 10.3390/vaccines9060583 (PMC8227098; doi:10.3390/vaccines9060583)
Supplement: Supplementary file 1 [file vaccines-09-00583-s001.zip › Document S3. Consent Form.pdf]

FORMULAIRE DE CONSENTEMENT - VOLET COMMUNAUTAIRE

NEOVAC 2 - Burkina Faso

Je soussignée, Mme ………………………………………………………………………………………… **accepte librement et volontairement** de participer à la recherche intitulée NEOVAC 2 - Burkina Faso avec mon enfant.

L'investigateur m'a expliqué les objectifs et les procédures de l'étude. J'ai eu la possibilité de poser toutes les questions se rapportant à cette étude et j'ai disposé d'un temps suffisant pour prendre ma décision.

J'ai été libre d'accepter tout comme de refuser cette étude et je peux à tout moment décider de ne plus y participer. Je peux également retirer mon consentement à tout moment sans avoir à me justifier. En cas de retrait de mon consentement, tous les échantillons ainsi que les données collectées pourront être détruits à ma demande. Si je me retire de l'étude et que les investigateurs de la recherche n'arrivent pas à me joindre, les données déjà recueillies nous concernant, mon enfant et moi, pendant ma participation à l'étude ainsi que mes échantillons biologiques ou ceux de mon enfant seront détruits.

J'ai bien compris que cette étude consiste en un **dépistage du virus de l'hépatite B** pour mon enfant et moi. **Dans certains cas la vaccination de mon enfant** contre l'hépatite B me sera proposée à la naissance.

J'ai bien noté que le présent consentement ne décharge pas le promoteur et l'investigateur de leurs responsabilités et je conserve tous mes droits garantis par la loi.

J'accepte l'utilisation de mes échantillons sanguins et ceux de mon enfant pour des recherches génétiques dans le cadre de cette étude ou pour des études ultérieures sur les hépatites virales.	❒OUI ❒ NON
J'accepte la conservation et l'utilisation de mes échantillons sanguins, de mes données et ceux de mon enfant pour des études ultérieures dans le domaine des hépatites virales.	❒OUI ❒ NON
J'accepte que les données individuelles obtenues dans le cadre de la recherche puissent, après avoir été codées, être versées dans des bases de données et mises à la disposition de la communauté scientifique académique et industrielle, selon un processus très strict d'accès, au sein de l'union européenne et en dehors.	❒OUI ❒ NON
J'accepte que les échantillons et les données associées, y compris génétiques, recueillis dans le cadre de cette étude puissent être conservés et partagés avec des partenaires académiques ou industriels, au sein de l'union européenne et en dehors (pays assurant le même niveau de protection de la vie privée qu'en Europe ou partenaires s'engageant à assurer ce haut niveau de protection).	❒OUI ❒ NON

Je peux, si je le souhaite, poser d'autres questions et être informé(e) des résultats globaux de l'étude en contactant le médecin signataire.

Date : Langue de l'entretien :

Signature du participant (ou empreinte digitale):

Nom et signature du témoin (ou empreinte digitale) si applicable:

Date :

Nom de l'investigateur :

Tél. : ________________________________- Mail : ______________________________@____________________

J'atteste avoir pleinement expliqué à la personne signataire les buts et les modalités de la recherche.

Signature :

Un exemplaire de la note d'information et le second volet de ce formulaire de consentement me seront remis. Le premier volet sera conservé par l'investigateur.
