# Supplementary material for: Impact of Introducing Hepatitis B Birth Dose Vaccines into the Infant Immunization Program in Burkina Faso: Study Protocol for a Stepped Wedge Cluster Randomized Trial (NéoVac Study)
Source: Vaccines (Basel). 2021 Jun 1;9(6):583. doi: 10.3390/vaccines9060583 (PMC8227098; doi:10.3390/vaccines9060583)
Supplement: Supplementary file 1 [file vaccines-09-00583-s001.zip › Document S4. Case Report Form.pdf]

Neovac Phase 2 - Burkina Faso

Evaluation de l'intégration d'une dose néonatale de vaccin contre l'hépatite B dans le calendrier vaccinal des enfants au Burkina Faso

CAHIER D'OBSERVATION

Protocole d'étude N°2017-083 ; version 8F du 12 mars 2020

Identification de la mère : |__|__| - |__|__|__|__|

Code CSPS (cf. liste des codes à la page suivante)	Numéro d'ordre de la participante (0001 pour la première inclusion dans chaque CSPS, etc.)

Promoteur :
Institut Pasteur
25 rue du Docteur Roux
75724 Paris Cedex 15

Promoteur délégué:
LAMIVAC
Avenue Konaté, 01 BP 112, Bobo-Dioulasso
+226 20 98 20 41

Investigateurs principaux :
Yusuke SHIMAKAWA (Institut Pasteur Paris)
Unité d'épidémiologie des maladies émergentes
tél. : +33 (0)1 40 61 38 87
yusuke.shimakawa@pasteur.fr

Muriel VRAY (Institut Pasteur Paris)
Unité d'épidémiologie des maladies émergentes
tél. :+33 (0)1 40 61 39 58
muriel.vray@pasteur.fr

Investigateurs locaux :
Abdoul TIENDREBEOGO (LAMIVAC)
tél. : +226 71 12 05 06
tiendraseric@gmail.com

Dramane KANIA (Centre Muraz)
tél. : +226 70 74 89 13
draka3703@yahoo.fr

Haoua TALL (AMP)
tél. : +226 20 98 20 41
htall@aamp.org

Liste des codes des CSPS impliqués dans l'étude

District	CSPS	Code CSPS
Dafra	Panamasso	**01**
	Kouentou	**02**
	Santidougou	**03**
	Léguema	**04**
	Kotédougou	**05**
	Yégueresso	**06**
	Baré	**07**
	Sogossagasso	**08**
Do	Kiri	**09**
	Peni	**10**
	Toussiana	**11**
	Tapoko	**12**
	Logofourousso	**13**
	Koumi	**14**
	Tiara	**15**
	Nasso	**16**
	Karangasso/Sambla	**17**
	Bouende	**18**
	Matourkou	**19**
	Dodougou	**20**
	Kouakouale	**21**
	Gna fongon	**22**
	Moussobadougou	**23**
	Ouolokoto	**24**

Instructions générales de remplissage

- ✓ Remplir les feuilles en utilisant un stylo à bille noir pour une bonne lisibilité.
- ✓ Ecrire en lettres capitales : |_N_|_E_|_O_|_V_|_A_|_C_|
- ✓ N'inscrire qu'un seul caractère par case et remplir toutes les cases : |_0_|_3_|,|_4_|_0_| kg
- ✓ Lorsque le résultat doit être reporté dans une case fermée, cochez la case appropriée :

  ☒ Oui ☐ Non

- ✓ Chaque erreur doit être barrée d'un trait, de manière à la laisser visible. La nouvelle donnée doit être écrite à côté en datant et paraphant. Ne pas utiliser de correcteur :

  ~~|_3_|_6_|~~ *32 (LS le 01/01/2020)*

- ✓ Si une date est totalement inconnue par la participante, merci de cocher « Ne sait pas ». Si une date partiellement connue, merci de remplir les cases manquantes avec la mention **XX** (par exemple, seule l'année est connue : XX/XX/2020 ou seul le jour est inconnu : XX/01/2020).
- ✓ En cas d'événement indésirable, quel que soit le moment de la déclaration, remplir le feuillet EI à la page 9 du cahier d'observation. Tous les événements indésirables graves (EIG) doivent être déclarés au promoteur via le formulaire de « déclaration des événements indésirables graves ».
- ✓ La fiche de « Bilan de fin d'étude » à la page 8 du cahier d'observation doit toujours être complétée à la fin du suivi, même si la mère et/ou l'enfant ne sont pas revenus à la visite des 9 mois.

Critères d'inclusion

1. Date de visite : |__|__|/|__|__|/|__|__|__|__| (jj/mm/aaaa)
2. Type de visite : ☐$_1$ CPN ☐$_2$ Accouchement<br>↳ 2.1. Si « CPN », indiquez le n° de CPN :<br>☐$_1$ CPN 1 ☐$_2$ CPN 2 ☐$_3$ CPN 3 ☐$_4$ CPN 4 ☐$_5$ CPN 5 ☐$_6$ CPN 6 ☐$_7$ CPN 7 ☐$_8$ CPN 8
3. Nom du CSPS visité : ____________________
4. Clinicien / Agent de santé : ____________________

**Critères d'inclusion**		
5. Femme enceinte ou femme qui vient d'accoucher	☐$_0$ Non	☐$_1$ Oui
6. Fréquentant un CSPS participant à l'étude pour une visite prénatale ou l'accouchement	☐$_0$ Non	☐$_1$ Oui
7. Résidente dans les aires sanitaires couvertes par les CSPSs ruraux des districts sanitaires de Dafra ou Do	☐$_0$ Non	☐$_1$ Oui
8. Formulaire de consentement éclairé signé après information de la femme	☐$_0$ Non	☐$_1$ Oui

↳ Si au moins l'un des critères est coché **NON**, la femme ne peut pas être incluse

**Critères de non inclusion**		
9. Participation à une autre recherche incompatible avec les modalités de protocole	☐$_0$ Non	☐$_1$ Oui
10. Toute condition de l'enfant ou de la mère qui, selon le professionnel de santé, est incompatible avec les examens réalisés pour l'étude	☐$_0$ Non	☐$_1$ Oui

↳ Si l'un des critères est coché **OUI**, la femme ne peut pas être incluse

**Résultat de l'inclusion**
11. Femme incluse dans l'étude : ☐$_1$ Oui ☐$_0$ Non

**Participation de la femme**
12. La femme a-t-elle déjà été incluse dans cette étude (NéoVac 2) pour une **grossesse précédente** :<br>☐$_0$ Non<br>☐$_1$ Oui<br>↳ 12.1. Si « Oui », indiquez son ancien identifiant : |__|__| - |__|__|__|__|__|

V1 – Visite d'inclusion

**Données sur la femme**
13. Village/hameau d'habitation : ____
14. CSPS de référence par rapport au village : __________________________________
15. N° d'ordre de la femme dans le registre de CPN : |__|__|__|__| / |__|__|__|__|__| ↳ 15.1. Si la femme a réalisé la/les CPN dans un autre CSPS, précisez le nom :<br>____
16. Date de naissance : |__|__|/|__|__|/|__|__|__|__| (jj/mm/aaaa) ☐9 Ne sait pas<br>↳ 16.1. Si « Ne sait pas », précisez l'âge approximatif : |__|__| ans
17. Situation matrimoniale :<br>☐1 Mariée monogame<br>☐2 Mariée polygame<br>☐3 Divorcée<br>☐4 Célibataire<br>☐5 Veuve
19. Ethnie :<br>☐1 Bobo<br>☐2 Tiéfo<br>☐3 Mossi<br>☐4 Dioula<br>☐5 Peuhl<br>☐6 Autre<br>↳ 19.1. Si « Autre », précisez : ____
20. Niveau d'instruction :<br>☐0 N'a pas été à l'école<br>☐1 Primaire<br>☐2 Secondaire<br>☐3 Supérieur
21. Gestité (nombre total de grossesses antérieures + actuelle) : |__|__| ; ☐9 Ne sait pas
22. Parité (nombre total d'accouchements antérieurs) : |__|__| ; ☐9 Ne sait pas
23. Date des dernières règles : |__|__|/|__|__|/|__|__|__|__| (jj/mm/aaaa) ☐9 Ne sait pas
24. Date probable d'accouchement : |__|__|/|__|__|/|__|__|__|__| (jj/mm/aaaa)<br>☐9 Ne sait pas ☐8 NA (pour les femmes qui sont incluses au moment de V2)

V2 – Visite d'accouchement ou post-accouchement

Pour les enfants qui sont mort-nés ou décédés après la naissance, merci de compléter ce formulaire (V2), puis allez directement au formulaire « Bilan de fin d'étude » (p.8)

25. Date de visite : |__|__|/|__|__|/|__|__|__|__| (jj/mm/aaaa)
26. Type de visite : ☐1 Accouchement ☐2 Consultation postnatale
27. Nom du CSPS visité : ____________________
28. Clinicien / Agent de santé : ____________________

Données sur les consultations prénatales

29. Nombre total de consultations prénatales réalisées durant la grossesse : |__| ; ☐9 Ne sait pas **(Si « 0 » allez à Q.32, sinon allez à Q.30)**

30. Dépistage VIH lors d'une consultation prénatale :

- ☐0 Fait et résultat négatif
- ☐1 Fait et résultat positif
- ☐2 Non fait
- ☐9 Ne sait pas

↳ 30.1. Si « Fait et résultat positif », est ce qu'un traitement antiviral a été initié durant la grossesse ?

- ☐1 Oui
- ☐0 Non
- ☐9 Ne sait pas

↳ 30.1.1. Si « Oui », quand : |__|__|/|__|__|/|__|__|__|__| (jj/mm/aaaa) ☐9 Ne sait pas

31. Dépistage VHB (l'hépatite B) lors d'une consultation prénatale :

- ☐0 Fait et résultat négatif
- ☐1 Fait et résultat positif
- ☐2 Non fait
- ☐9 Ne sait pas

↳ 31.1. Si « Fait et résultat positif », est ce qu'un traitement antiviral a été initié durant la grossesse ?

- ☐1 Oui
- ☐0 Non
- ☐9 Ne sait pas

↳ 31.1.1. Si « Oui », quand : |__|__|/|__|__|/|__|__|__|__| (jj/mm/aaaa) ☐9 Ne sait pas

Données sur l'accouchement

32. Date de l'accouchement: |__|__|/|__|__|/|__|__|__|__| (jj/mm/aaaa)

33. Lieu de l'accouchement :

☐1 Dans un CSPS (précisez le nom : ________________________________
et le n° d'ordre de la femme dans le registre d'accouchement : _______________)
☐2 Dans un CMA (précisez le nom : ________________________________)
☐3 Dans un CHU (précisez le nom : ________________________________)
☐4 Dans une structure privée (précisez le nom : ________________________)
☐5 Au domicile de la femme
☐6 Au domicile d'une autre personne
☐7 Sur le lieu de travail de la femme
☐8 Chez un tradipraticien (guérisseur)
☐9 Chez une accoucheuse traditionnelle
☐10 Sur la route vers une structure de santé
☐11 Autre (précisez : ____)

34. Intervention réalisée durant l'accouchement :

34.1. Césarienne	☐0 Non	☐1 Oui	☐9 Ne sait pas
34.2. Ventouse	☐0 Non	☐1 Oui	☐9 Ne sait pas
34.3. Version	☐0 Non	☐1 Oui	☐9 Ne sait pas
34.4. Episiotomie	☐0 Non	☐1 Oui	☐9 Ne sait pas

35. Multiplicité :

☐1 Simple
☐2 Jumeaux
☐3 Triplés

Données sur le 1er enfant de cet accouchement **(si plusieurs enfants sont nés au moment de l'accouchement, merci de compléter un ou plusieurs formulaires supplémentaires en fonction du nombre d'enfants et de les coller à la suite de ce cahier d'observation)**

36. Identifiant de l'enfant n°1 : |__|__| - |__|__|__|__| - 1

Identifiant de la mère

37. Heure de naissance : |__|__| H |__|__| (format 24h (E.G., 16h00)) ☐9 Ne sait pas

38. Prématuré (avant 37 semaines révolues d'aménorrhée) :

☐1 Oui
☐0 Non
☐9 Ne sait pas

39. L'enfant n°1 est-il mort-né ?

☐1 Oui
☐0 Non

Si « Oui » allez directement au formulaire « Bilan de fin d'étude » (page 8)

40. Poids de naissance : |__|__|__|__| g

41. Taille de naissance : |__|__| , |__| cm

42. Périmètre crânien : |__|__| , |__| cm

43. Périmètre thoracique : |__|__| , |__| cm

44. Sexe de l'enfant :

☐1 Masculin
☐2 Féminin

45. APGAR : 1 min : |__|__| 5 min : |__|__| 10 min : |__|__| ☐1 NA

Données sur l'intervention (vaccination à la naissance contre l'hépatite B)

46. L'injection du vaccin contre l'hépatite B à la naissance a-t-elle eu lieu pour l'enfant n°1 ?

☐1 Oui

↳ Si « Oui », précisez :

46.1.1. Date de l'injection : |__|__|/|__|__|/|__|__|__|__| (jj/mm/aaaa)

46.1.2. Heure de l'injection : |__|__| H |__|__|

46.1.3. Numéro du lot : |__|__|__|__|__|__|__|__|

☐0 Non

↳ 46.2.1. Si « Non », pourquoi ?

☐1 Le vaccin n'était pas disponible dans le CSPS
☐2 L'enfant est revenu après 8 semaines de vie
☐3 Refus de vacciner

↳ 46.2.1.1. Si « Refus de vacciner », qui a refusé ? _______________

____

☐4 Autre

↳ 46.2.1.2. Si « Autre », précisez : _______________________

____

47. L'enfant n°1 a-t-il reçu des traitements depuis la naissance ?

☐1 Oui
☐0 Non

<u>**Si « Oui », merci de compléter le formulaire des traitements concomitants (page 10)**</u>

V3 - Visite de dépistage du couple mère-enfant

Pour les enfants qui sont décédés, merci de compléter les questions Q.51 à Q.53 de ce formulaire (V3), puis allez directement au formulaire « Bilan de fin d'étude » (p.8)

48. Date de visite : |__|__|/|__|__|/|__|__|__|__| (jj/mm/aaaa)
49. Nom du CSPS visité : ____
50. Clinicien / Agent de santé : ____

**Données à 9 mois sur le 1er enfant de l'accouchement**			
**51. Vaccination du 1er enfant de l'accouchement (cf. carnet/carte de vaccination de l'enfant)**			
**Age**	**Vaccin**	**Vaccination**	**Date (jj/mm/aaaa)**
Dès la naissance	HepB 0	☐1 Oui ☐0 Non ☐9 Ne sait pas	|__|__|/|__|__|/|__|__|__|__|
	VPO 0	☐1 Oui ☐0 Non ☐9 Ne sait pas	|__|__|/|__|__|/|__|__|__|__|
	BCG	☐1 Oui ☐0 Non ☐9 Ne sait pas	|__|__|/|__|__|/|__|__|__|__|
2 mois	Penta 1	☐1 Oui ☐0 Non ☐9 Ne sait pas	|__|__|/|__|__|/|__|__|__|__|
	VPO 1 (Polio 1)	☐1 Oui ☐0 Non ☐9 Ne sait pas	|__|__|/|__|__|/|__|__|__|__|
	Pneumo 1	☐1 Oui ☐0 Non ☐9 Ne sait pas	|__|__|/|__|__|/|__|__|__|__|
	Rota 1	☐1 Oui ☐0 Non ☐9 Ne sait pas	|__|__|/|__|__|/|__|__|__|__|
3 mois	Penta 2	☐1 Oui ☐0 Non ☐9 Ne sait pas	|__|__|/|__|__|/|__|__|__|__|
	VPO 2 (Polio 2)	☐1 Oui ☐0 Non ☐9 Ne sait pas	|__|__|/|__|__|/|__|__|__|__|
	Pneumo 2	☐1 Oui ☐0 Non ☐9 Ne sait pas	|__|__|/|__|__|/|__|__|__|__|
	Rota 2	☐1 Oui ☐0 Non ☐9 Ne sait pas	|__|__|/|__|__|/|__|__|__|__|
4 mois	Penta 3	☐1 Oui ☐0 Non ☐9 Ne sait pas	|__|__|/|__|__|/|__|__|__|__|
	VPO 3 (Polio 3)	☐1 Oui ☐0 Non ☐9 Ne sait pas	|__|__|/|__|__|/|__|__|__|__|
	Pneumo 3	☐1 Oui ☐0 Non ☐9 Ne sait pas	|__|__|/|__|__|/|__|__|__|__|
	Rota 3	☐1 Oui ☐0 Non ☐9 Ne sait pas	|__|__|/|__|__|/|__|__|__|__|
	VPI	☐1 Oui ☐0 Non ☐9 Ne sait pas	|__|__|/|__|__|/|__|__|__|__|

Dépistage AgHBs des couples mère-enfant

52. Résultat du test rapide AgHBs **pour la mère biologique** : **(si le résultat est indéterminé, merci de refaire le test jusqu'à ce que le résultat apparaisse)**

☐0 Négatif
☐1 Positif

↳ Si « Positif », précisez :

52.1. Prélèvement veineux de 8 mL sur tube sec effectué : ☐1 Oui ☐0 Non

52.2. Date de rendez-vous au CMA : |__|__|/|__|__|/|__|__|__|__| (jj/mm/aaaa)

53. La collection sur papier buvard a-t-elle été réalisée **pour la mère biologique** ?

☐1 Oui
☐0 Non

↳ 53.1. Si « Non », pourquoi :

____

____

54. Résultat du test rapide AgHBs **pour le 1er enfant** : **(si le résultat est indéterminé, merci de refaire le test jusqu'à ce que le résultat apparaisse)**

☐0 Négatif
☐1 Positif

↳ Si « Positif », précisez :

54.1. Prélèvement veineux de 4 mL sur tube sec effectué : ☐1 Oui ☐0 Non

54.2. Date de rendez-vous au CMA : |__|__|/|__|__|/|__|__|__|__| (jj/mm/aaaa)

55. La collection sur papier buvard a-t-elle été réalisée **pour le 1er enfant** ?

☐1 Oui
☐0 Non

↳ 55.1. Si « Non », pourquoi :

____

____

Événements indésirables

56. Des événements indésirables sont-ils survenus pour la mère ou l'enfant n°1 depuis la naissance de l'enfant ?

☐1 Oui
☐0 Non

Si « Oui », merci de compléter le formulaire des événements indésirables (page 9)

Traitements concomitants

57. Des traitements concomitants ont-ils été reçus par la mère ou l'enfant n°1 depuis la naissance de l'enfant ?

☐1 Oui
☐0 Non

Si « Oui », merci de compléter le formulaire des traitements concomitants (page 10)

Bilan fin d'étude

Condition fin d'étude

58. Toutes les visites ont été effectuées pour la mère et l'enfant n°1 :

☐1 Oui
☐0 Non

↳ 58.1. Si « Non », pourquoi :

☐1 Enfant n°1 décédé

↳ 58.1.1.1. Condition du décès ☐1 Fausse couche
☐2 Mort-né
☐3 Décédé après la naissance

↳ 58.1.1.2. Date de décès : |__|__|/|__|__|/|__|__|__|__| (jj/mm/aaaa) ☐9 Ne sait pas

↳ 58.1.1.3. Convocation de la mère pour prélèvement : ☐1 Oui ☐0 Non

↳ 58.1.1.4. La mère a-t-elle été prélevée : ☐1 Oui ☐0 Non

<u>Si la mère a été prélevée, merci de compléter les questions Q.52 et Q.53 à la page 7</u>

☐2 Mère décédée

↳ 58.1.2.1. Date de décès : |__|__|/|__|__|/|__|__|__|__| (jj/mm/aaaa) ☐9 Ne sait pas

↳ 58.1.2.2. L'enfant a-t-il été convoqué pour le prélèvement : ☐1 Oui ☐0 Non

↳ 58.1.2.3. L'enfant a-t-il été prélevé : ☐1 Oui ☐0 Non

<u>Si l'enfant a été prélevé, merci de compléter les questions Q.54 et Q.55 à la page 7</u>

☐3 Autre

↳ 58.1.3. Si « Autre », précisez :

☐1 Refus

↳ 58.1.3.1. Qui a refusé ? ______________________________________

↳ 58.1.3.2. Précisez la raison du refus : ___________________________

____

↳ 58.1.3.3. Autorisation de conserver les données collectées sur le couple mère-enfant pour l'analyse de l'étude ? ☐1 Oui ☐0 Non

☐2 Déménagement
☐3 Autre

↳ 58.1.3.4. Si « Autre », précisez :

____

____

☐4 Aucune information concernant la mère (perdue de vue)

Evénement indésirable

Événement	Personne concernée	Date de survenue	Intensité	Action	Evolution	Imputabilité	Date de fin	E.I. grave (**)
	0=Mère<br>1=Enfant n°1<br>2=Enfant n°2<br>3=Enfant n°3	JJ/MM/AAAA	1=Faible<br>2=Moyenne<br>3=Forte	1=Aucune<br>2=Traitement(s) symptomatique(s) seul(s) (*)<br>3=Hospitalisation (**)<br>4=Autre (préciser en commentaire)	1=Guérison<br>2=Non encore rétabli<br>3=Séquelles (**)<br>4=Décès (**)<br>5=Inconnue	0=Non lié à la recherche<br>1=Lié à la recherche	JJ/MM/AAAA	0=Non<br>1=Oui
1.	|__|	_ _ /_ _ / _ _ _ _	|__|	|__|	|__|	|__|	_ _ /_ _ / _ _ _ _	|__|
2.	|__|	_ _ /_ _ / _ _ _ _	|__|	|__|	|__|	|__|	_ _ /_ _ / _ _ _ _	|__|
3.	|__|	_ _ /_ _ / _ _ _ _	|__|	|__|	|__|	|__|	_ _ /_ _ / _ _ _ _	|__|
4.	|__|	_ _ /_ _ / _ _ _ _	|__|	|__|	|__|	|__|	_ _ /_ _ / _ _ _ _	|__|
5.	|__|	_ _ /_ _ / _ _ _ _	|__|	|__|	|__|	|__|	_ _ /_ _ / _ _ _ _	|__|
6.	|__|	_ _ /_ _ / _ _ _ _	|__|	|__|	|__|	|__|	_ _ /_ _ / _ _ _ _	|__|
7.	|__|	_ _ /_ _ / _ _ _ _	|__|	|__|	|__|	|__|	_ _ /_ _ / _ _ _ _	|__|
8.	|__|	_ _ /_ _ / _ _ _ _	|__|	|__|	|__|	|__|	_ _ /_ _ / _ _ _ _	|__|
9.	|__|	_ _ /_ _ / _ _ _ _	|__|	|__|	|__|	|__|	_ _ /_ _ / _ _ _ _	|__|
10.	|__|	_ _ /_ _ / _ _ _ _	|__|	|__|	|__|	|__|	_ _ /_ _ / _ _ _ _	|__|

(*) En cas de traitement(s) symptomatique(s), compléter la page des traitements concomitants.

(**) En cas d'événements indésirables grave, prévenir immédiatement l'ARC du LAMIVAC par téléphone au 72 23 92 85/71 12 05 06 ou par email fsawadogo@aamp.org au moyen du formulaire de « déclaration des événements indésirables graves ».

Traitement concomitant

Nom générique ou commercial	Personne concernée	Date de début	Posologie	Voie	Indication	Date de fin
	0=Mère<br>1=Enfant n°1<br>2=Enfant n°2<br>3=Enfant n°3	JJ/MM/AAAA		1=Orale<br>2=Intramusculaire<br>3=Intraveineuse<br>4=Sous cutanée<br>5=Transdermique<br>6=Rectale<br>7=Autre		JJ/MM/AAAA
1.	|__|	_ _ /_ _ / _ _ _ _		|__|		_ _ /_ _ / _ _ _ _
2.	|__|	_ _ /_ _ / _ _ _ _		|__|		_ _ /_ _ / _ _ _ _
3.	|__|	_ _ /_ _ / _ _ _ _		|__|		_ _ /_ _ / _ _ _ _
4.	|__|	_ _ /_ _ / _ _ _ _		|__|		_ _ /_ _ / _ _ _ _
5.	|__|	_ _ /_ _ / _ _ _ _		|__|		_ _ /_ _ / _ _ _ _
6.	|__|	_ _ /_ _ / _ _ _ _		|__|		_ _ /_ _ / _ _ _ _
7.	|__|	_ _ /_ _ / _ _ _ _		|__|		_ _ /_ _ / _ _ _ _
8.	|__|	_ _ /_ _ / _ _ _ _		|__|		_ _ /_ _ / _ _ _ _
9.	|__|	_ _ /_ _ / _ _ _ _		|__|		_ _ /_ _ / _ _ _ _
10.	|__|	_ _ /_ _ / _ _ _ _		|__|		_ _ /_ _ / _ _ _ _
11.	|__|	_ _ /_ _ / _ _ _ _		|__|		_ _ /_ _ / _ _ _ _
12.	|__|	_ _ /_ _ / _ _ _ _		|__|		_ _ /_ _ / _ _ _ _
